# Circular—Mortality Statistics in the Twelfth Census

**Published:** 1899-11

**Authors:** William R. Merriam

**Affiliations:** Director of the Census


### Abstracts and Selections.

### Circular—Mortality Statistics in the Twelfth Census.

This circular is issued for the information of all persons officially engaged or interested in the collection and registration of statistics of mortality within any given registration area in the United States, in the hope of bringing about a larger measure of uniformity of statement of the facts required by law to be included in the returns of deaths made to the Census Office for the year 1900.

### PROVISIONS OF THE CENSUS ACT.

The seventh section of the act entitled "An act to provide for taking the twelfth and subsequent censuses," approved March 3, 1899, declares that "the Twelfth Census shall be restricted to inquiries relating to the population, to mortality, to the products of agriculture and of manufacture and mechanical establishments. * * * The mortality schedules shall comprehend for each decedent the name, sex, color, age, conjugal condition, place of birth, and birth place of parents, occupation, cause and date of death, and, if born within the census year, the date of birth. The form and arrangement of the schedule and the specific questions necessary to secure the information required shall be in the discretion of the Director. * * * In cities or States where an official registration of deaths is maintained, the Director of the Census may, in his discretion, withhold the mortality schedule from the several enumerators within such cities or States, and may obtain the information required by this act through official records."

REGISTRATION AREAS.

The laws governing the official registration of vital and mortality statistics in the several States are not uniform. In some States the law provides for a general system of registration and the making of returns of births and deaths to a central office, but in others the registration is purely local, being confined to certain towns or cities. Complete and accurate knowledge of the methods adopted and of the extent to which different systems are followed and in force locally by States or minor civil divisions, is the first desideratum in the formulation of a scheme of uniform statistics of mortality covering the entire country. An attempt has already been made to secure partial information as to this point by means of letters addressed to State officials in each State known to have a general registration law, and to the health officers of all cities possessing a population in 1890 of not less than five thousand (5000). The response to this inquiry has been most gratifying, but all persons into whose hands this circular may come are urgently requested to forward to the Chief Statistician of the Census Office for Vital Statistics (Mr. William A. King) immediately, if they have not yet so done, whatever documents, reports, blanks, or other published or unpublished material may be at their disposal for this purpose, which will throw additional light upon the existing organization and scope of official registration of births and deaths in the United States, or in any State, Territory, county, town, or city thereof. For this a franked return envelope is enclosed, which may be used for correspondence, and a Census label to be attached to packages of books and pamphlets. Official communications to this office require no postage stamps, and, in order to save expense, packages sent to it should always be forwarded by mail, and not by express.

DISCRETION OF THE DIRECTOR.

It will be observed that the Director of the Census has, under the law, discretionary power to collect statistics of mortality in either of two ways—by the enumerators appointed to make returns of the population or by withholding the mortality schedules from the enumerators and procuring the desired information from official records, and that the selection of registration areas in which to withhold these schedules from the enumerators is also at his discretion.

It is comparatively an easy matter to secure statistics of the living population though the Census enumerators; but experience has demonstrated the fact that they fail to report more than 50 or 60 per cent. of the deaths occurring in the country. The reason for this failure is plain: The living population is returned as of a single day, the first of June, and the enumeration is completed within thirty days from that date, but the return of deaths covers a period of twelve months. The percentage of omission is, as might be expected, least in the months immediately preceding the enumeration, and greatest in the months further removed in point of time from the inquiry.

The Census returns, therefore, obtained by enumerators, furnish no basis for a computation of death rates per thousand of the population in any State, or in any more restricted population area.

In view of these facts and considerations, the Director of the Census will make the largest practicable use of the discretion conferred upon him by law. Attention is, however, called to the fact that the census law confers no discretion upon the Director with reference to the substance of the inquiry, which must cover, for every death reported, (1) the decedent's name; (2) sex; (3) color; (4) age; (5) conjugal condition; (6) place of birth; (7) also the birthplace of each parent; (8) occupation; (9) cause of death; (10) date of death, and, if born within the census year; (11) date of birth.

The value to this office of official records which do not cover, with sufficient detail, all of the points included in the prescribed mortality schedule is, of course, impaired in proportion to the number and importance of the items omitted. The value of the Census report on mortality to students and to the public will be correspondingly less, if the omission of any item diminishes the total registration area and the aggregate number of cases reported for any particular inquiry.

IMPORTANCE OF UNIFORMITY.

In order to the statistical tabulation of any group of facts, uniformity of statement in the returns is essential. Otherwise comparisons are rendered impossible. This uniformity in the matter of mortality returns may be assumed to be secured, for each State, by the act governing the registration; but in a national report it is indispensable that there should be uniformity in the returns made by the several States or subdivisions of a State. Uniformity can be secured by confiding the mortality schedules to the enumerators, but, as has been shown, only at the cost of completeness and accuracy. Unless coöperation between the States can be effected by mutual consent and agreement, therefore, the only alternative is between uniformity without completeness and a complete enumeration of deaths within certain registration areas without uniformity or completeness of detail. The question is whether it is not possible to persuade the local officials in charge of the registration to modify the forms of their returns, so as to make them conform to the requirements of the Census Act, and thus secure both uniformity and completeness in the report to be published by this office.

The benefit to science which would result from uniform statistics of mortality for all the States is so apparent as to need no argument or elucidation. It seems to be conceded by all registration officers with whom correspondence upon the subject has been had. From the point of view of this office, the objective end sought in the study of the population is to determine its natural law of growth, as controlled by births, deaths, immigration, and emigration. A complete return of deaths is essential to the solution of the problem thus presented. No such return can be obtained by the agency of the enumeration, nor can it be obtained from official records, unless the officers in charge will agree to follow the lead of Congress and furnish, for use in the Census, full replies to all the inquiries contained in the mortality schedule. This, of course, they can not do without such modification of the forms of return now in local use as may be essential to the attainment of the desired result. In order to bring the local records and the Census Act into harmony with each other, it is the local records which must be adapted to the general law, since the Census Act can not be amended to confrom to the varying local requirements and practice.

THE CENSUS INQUIRIES.

Each of the inquiries embraced in the mortality schedule of the Census has a definite purpose, and the information supplied by the replies to the several questions asked constitutes an important factor in the compilation of certain tables which it is proposed to include in the final Census report. This is shown by the following explanatory notes relative to the several inquiries:

1. Name, in full.

2. Color; White, Black (Negro or Negro descent), Indian, Chinese, and Japanese.—This term (Color) includes Race, so far as the Census takes note of racial distinctions. Each constitutes a certain distinct class for which certain tables will be compiled. The degree of color of Negroes, as indicated by the words "Mulatto, Quadroon," etc., which was asked in the last Census, will not be asked in 1900, but all these details of color will be included under the general term "Black."

3. Sex; Male, Female.—The sex of each person should be positively stated. The work of compilation is retarded, if sex is left to be inferred from the given name, or from the use made in the return of the personal pronouns "he" and "she."

4. Conjugal Condition; Single, Married, Widowed, Divorced.—Very few of the certificates or returns in local use include any specification of "Divorced," and in many of them the only distinction made is between "Married" and "Single." The "Widowed" and "Divorced" may, therefore, be called either "Single" or "Married," according to the point of view, and, when so reported, can not be separated. In the computation of mortality, and to show the influence of conjugal condition upon the death rates due to certain causes, the distinctions indicated are equally important, and should be carefully maintained.

5. Date of Death; Year, Month, and Day of month.

6. Date of Birth.—This question occurs in some of the forms of return, of which copies have been forwarded to this office. It is of importance as a check upon the statements made as to age, particularly in the case of children. (See also "Age.")

7. Age; in Years, Months, and Days.—When age is called for, without the exactness required by these details, this question may be construed as referring to the person's age at the last birthday, the next birthday, or the nearest birthday. An exact statement of age is very important, especially in the case of children, because the correct computation of infantile mortality depends upon it, and because also the computation of life tables involves the work of construction in which slight errors in the statement of proportion and death rates of children are magnified when carried forward in the subsequent processes of calculation.

8. Occupation.—The effect of "Occupation" must necessarily be taken into consideration in any comprehensive mortality statistics.

The precise instructions given to the Census enumerators as to the description and classification of occupations can not be applied to, and their observance secured by, the physicians, undertakers, and others who report deaths to the registration officers. The general principle is to bear in mind what labor the deceased actually performed, without regard to the place or the person for whom he worked. Care should be taken to express the occupation in such a way as to prevent it from being confounded with other occupations, and it is preferable to give too much detail rather than too little.

9. Place of Birth.—If born in the United States, give the name of State or Territory; if of foreign birth, the name of country. Some forms of return in local use call for "Nationality," and, when this is used, the State or country of birth should also be given. (See Birthplace of Mother.)

10. Birthplace of Father.—Same as above.

11. Birthplace of Mother.—Same as above. The distinctions of sex, color, and general nativity run through all of the compilations. The birthplaces of parents are necessary in order to classify the deaths by parental nativity. The proportion of persons of foreign parentage is so large, and the difference in the death rates so considerable, that this becomes a very important factor. The "Birthplace of Mother," in particular, is extensively used, as best indicating the influence of race characteristics and inherited tendencies.

12. Disease, or Cause of Death.—This point is usually covered in a satisfactory manner in the official registration records.

13. Place of Death.—If in a city, the name of the street and the number of the house should be specified; the number of the ward should be stated also, and the ward designation verified by the registrars. In certain cities the data will be compiled in this office by "wards," and in others by "sanitary districts," which are subdivisions made by this office in accordance with local topography, drainage and sewerage, buildings, population characteristics, and general sanitary conditions. The precise location of the place of each death is necessary in order to credit it to the proper ward or sanitary district. If in a hospital or other institution, the name and location of the institution should be given, also the length of time the deceased was an inmate thereof, and the previous residence, in order that the death may be charged to the locality to which it is due and not to the neighborhood in which the institution happens to be situated. It would, obviously, be an error to charge a healthy sanitary district with all deaths occurring in some large hospital or institution receiving patients from other sections of the city.

14. Late Residence; in the City or State.—This is necessary to distinguish transients from residents and to permit transfer to the proper localities when such transfer should be made.

A precise answer should be furnished, so far as possible, to each of the foregoing questions, omitting none. All of them are required by the Census schedule, and every one of them is essential to the complete analysis and discussion of mortality statistics for the whole country.

DEPLORABLE EXISTING LACK OF UNIFORMITY.

Reference has been made to correspondence already had with registration officials. It is certainly not the province of the Census Office to criticise the legislation or administration of States or municipal corporations over which it has no jurisdiction; nor it is the purpose of this circular to cast any reflection upon arrangements which are satisfactory to the authorities responsible for instituting them and which meet the supposed local needs of particular communities. The most casual inspection of the forms and records now in use, however, in different cities, towns, and States will convince anyone that the differences between them in detail are very great.

The inquiries most commonly omitted from the returns of death required by law or by some municipal ordinance are those relating to the birthplace of parents, to occupation, and to conjugal condition. Where these questions are included in the blank form of return, they are those more frequently unanswered. Registration areas in which this information is furnished and those in which it is withheld can not be properly included in the same tables, for the purpose of comparison, or even of securing correct total additions. Two of the principal ends sought in statistical research are defeated by this unnecessary and objectionable variation in the form of official returns. Beyond this it should be remembered that death rates are percentages. Thev are obtained by dividing the number of deaths in a given area, with precise limits, by the population in the same area. The area must be the same in every instance, or the calculation is vitiated. No one unfamiliar by actual experience with Census work can form any correct notion of the amount of additional and needless work entailed upon this office, when it is compelled, in order to avoid drawing unwarranted conclusions, to make distinctions in the population of certain areas of registration corresponding to the distinctions made in the returns of deaths upon dissimilar bases, and for this purpose to have recourse to the original population schedules.

With regard to the return of death certificates to a central office, three distinct plans are followed in different localities: (1) the original certificates may themselves be transmitted; or (2) the originals are kept and certified copies of them are forwarded instead; or (3) the State Board of Health supplies sheets upon which a transcript of the originals is made. Experience has shown, and actual comparison of the copies and of the originals proves, that, in many instances where copies are accepted in lieu of the originals, information is furnished by the original certificate which does not appear upon the transcript.

It is a common practice to record death certificates, when received, upon the pages of a book or register kept for that purpose for convenient reference, after which the certificates are disposed of in various ways. This practice no doubt subserves a useful end, and the record kept is probably sufficient for all local demands, but the remark just made applies here also: The book does not in all cases contain information which was supplied by the original certificate. The local officers, because of the greater ease of copying, may insist upon furnishing the transcript asked by the Census Office from the book; and data accepted on the basis of the facts called for by the certificate may thus be supplied in a less complete form from the abbreviated record.

In some cases the original certificates, after they have been copied, are destroyed.

An element of uncertainty as to the statistical result is imported into the return, wherever any of the space provided upon the blank form is left without an entry, as, for instance, where the return calls for a statement of color "if other than white." The color should be stated, otherwise it is unknown. Wherever a correct answer is impossible, the word "unknown" should be written, but the use of this word should be restricted within the narrowest practicable limits.

The variations in practice in cities are even greater than in States. There are almost as many different forms of certificates of death as there are cities; and in many places the local authorities do not insist upon replies to certain questions included in the blank return. The proper rule to adopt is to refuse to accept, or to send back for correction, all improperly filled certificates.

Promptness in making returns is of the highest importance. In some States the local registrars are required to forward all returns to the State board as often as once in every month, in other States they are forwarded only once in a year, and in some cases the local registrars are even granted a delay of six months after the close of the official year before making their official return.

The circumstances and conditions of which incomplete mention has just been made, and others of similar import, must affect the use to be made of the discretionary power conferred upon the Director of the Census to "withhold the mortality schedule from the enumerators and obtain the information required through official records." In every instance it will be necessary to form a correct judgment of the value of the official record proposed to be consulted, before deciding whether a fuller and more satisfactory reply to the questions named in the Census Act can not be secured through the agency of the enumerators.

(The specimen "Form" for returns of a death, submitted in this circular is omitted. It can be had upon application to the Census Bureau.—Ed.)

The foregoing form is suggestive merely. It covers the inquiries prescribed by the Census Act, with such details as will enable this office in tabulating results to classify cases and distinguish between deaths of residents and of non-residents, and to distribute the deaths occurring in hospitals or other institutions. Any additional information required for local purposes can readily be provided for upon the same blank. In the form in which it is here presented it is applicable in registration States, where the local returns are collected in a central office; but it can be equally well adapted to cities, in other States, by a slight change in the heading.

### STILLBIRTHS.

Returns of stillbirths will be required, where they are of record; but in view of the fact that stillbirths are not reported in many registration areas, they will be excluded from the general compilation and separately tabulated.

### SUGGESTIONS INVITED.

All persons to whom this circular may be mailed are requested to acknowledge its receipt, and, in so doing, to express an opinion as to the desirability and practicability of taking advantage of this opportunity to inaugurate a uniform scheme of mortality registration, under the lead of the General Government, represented by the Census Office; also, to say precisely how much co-operation may be expected from the State or local board represented by the writer.

The field of official registration of vital statistics is rapidly enlarging, and assurances of co-operation have already been given in many quarters. It is hoped that practically universal co-operation may be brought about at this time. To afford opportunity for the movement thus inaugurated to mature and bear fruit, it is proposed to apply to the officials in charge of registration records for information covering the calendar year, January 1 to December 31, 1900, instead of the twelve months immediately preceding June 30, 1900, the date of taking the Census. This change will have the further advantage that the population on the 1st of June, 1900, will more closely approximate the mean population for the calendar year, and, therefore, greater accuracy can be attained in the calculation of death rates by the adoption of the calendar instead of the census year, as the period for investigation.

It will contribute largely to the end sought, if the medical journals of the United States will discuss the questions herein raised in their editorial columns, and open their pages to correspondence on the subject by medical men; also if it can be made the theme of discussion in medical conventions. Marked copies of all printed articles relating to it should be sent to this office, and will not only be gratefully received and acknowledged, but carefully considered.

WILLIAM R. MERRIAM,
Director of the Census.

